# RNAMethPre: A Web Server for the Prediction and Query of mRNA m^6^A Sites

**DOI:** 10.1371/journal.pone.0162707

**Published:** 2016-10-10

**Authors:** Shunian Xiang, Ke Liu, Zhangming Yan, Yaou Zhang, Zhirong Sun

**Affiliations:** 1 MOE Key Laboratory of Bioinformatics, School of Life Sciences, Tsinghua University, Beijing, 100084, P. R. China; 2 Key Lab in Healthy Science and Technology, Division of Life Science, Graduate School at Shenzhen, Tsinghua University, Beijing, 518055, P. R. China; 3 Department of Statistics, University of California, Berkeley, California, 94720, United States of America; Huazhong University of Science and Technology, CHINA

## Abstract

*N*^6^-Methyladenosine (m^6^A) is the most common mRNA modification; it occurs in a wide range of taxon and is associated with many key biological processes. High-throughput experiments have identified m^6^A-peaks and sites across the transcriptome, but studies of m^6^A sites at the transcriptome-wide scale are limited to a few species and tissue types. Therefore, the computational prediction of mRNA m^6^A sites has become an important strategy. In this study, we integrated multiple features of mRNA (flanking sequences, local secondary structure information, and relative position information) and trained a SVM classifier to predict m^6^A sites in mammalian mRNA sequences. Our method achieves ideal performance in both cross-validation tests and rigorous independent dataset tests. The server also provides a comprehensive database of predicted transcriptome-wide m^6^A sites and curated m^6^A-seq peaks from the literature for both human and mouse, and these can be queried and visualized in a genome browser. The RNAMethPre web server provides a user-friendly tool for the prediction and query of mRNA m^6^A sites, which is freely accessible for public use at http://bioinfo.tsinghua.edu.cn/RNAMethPre/index.html.

## Introduction

*N*^6^-Methylated-adenosine (m^6^A) is the most common and abundant modification on RNA molecules and exists in various species [1]. Although it was first detected in poly-A mRNA about 4 decades ago [2], m^6^A has not been characterized until the recent development of a transcriptome-wide mapping method called m^6^A-seq or MeRIP-seq [3, 4]. Using this method, the first m^6^A profiles were obtained for human and mouse. Based on mapping data, each mRNA contains, on average, 3–5 m^6^A modifications within DRACH (where D = A, G or U; R = A or G; H = A, C or U) consensus sequences, which are located in the coding sequence, UTRs, and introns of mRNAs and are especially enriched around stop codons [3–5]. Subsequent studies have found that m^6^A plays important roles in various biological processes, including splicing [4], mRNA stability [6], miRNA biogenesis [7], circadian clock regulation [8], and the developmental regulation of mammalian embryonic stem cells [9].

The m^6^A-seq method generates 100–200-nt peaks, but cannot be used to locate specific sites of m^6^A modification [3, 4]. Regev et al. improved the method and generated the transcriptome-wide m^6^A profile for yeast at nearly single-base resolution [10]. Using the high-resolution yeast dataset, two m^6^A site prediction servers, m6Apred [11] and iRNA-Methyl [12], have been developed based on different features. Both prediction methods exhibit acceptable performance in cross-validation tests using yeast datasets, but they cannot be applied to other taxon. More recently, Linder et al. developed a new method termed miCLIP and produced a single-nucleotide resolution map of the m^6^A sites across the human and mouse transcriptomes [13]. The availability of accurate m^6^A site datasets led to the first mammalian m^6^A site prediction server, SRAMP, established by Zhou et al. SRAMP employs a random forest machine learning framework using only sequence-derived features, including a positional binary encoding of flanking nucleotide sequences, the K-nearest neighbor (KNN), and the nucleotide pair spectrum [14]. The predictor achieved good performance in full transcript mode. However, there is still room for improvement, e.g., the performance in mature mRNA mode can be enhanced and increasingly user-friendly interfaces can be developed.

Here, we developed a user-friendly web server for m^6^A site prediction and query, named RNAMethPre, for human, mouse, and mammal, broadly. A support vector machine (SVM) was used to build the model with all features combined in a single classifier. The predictors achieved ideal performance not only in full transcript mode, but also in mature mRNA mode. Users can submit one or more mRNA sequences for prediction and tasks are completed rapidly owing to the high efficiency of our SVM method. To enhance the web-server, we applied the SVM model to predict all human and mouse transcripts. Experimental m^6^A-seq peaks and sites reported in previous publications were collected. As a result, a comprehensive database of transcriptome-wide m^6^A sites from prediction results and experimental data was created and integrated into the web server to provide a query service. Furthermore, a genome browser was established to visualize the m^6^A sites across the whole transcriptome.

## Methods

### Datasets

#### Positive dataset

Single-base resolution m^6^A site data generated using the miCLIP approach were collected from the literature [13, 15]. Most of the m^6^A sites were located in the consensus motif DRA*CH (where D denotes A, G, or U, R denotes A or G, A* denotes methylated A, and H denotes A, C, or U), consistent with previous m^6^A maps obtained using m^6^A-seq [4, 13]. Methylated adenosines within DRACH motifs were kept as positive samples. These sites were mapped to the longest isoforms of Ensembl coding genes (using the hg19 and mm9 assemblies). The resulting positive dataset contained 39396 human m^6^A sites and 30320 mouse m^6^A sites in mature mRNAs. A part of the m^6^A sites were not mapped to mature mRNAs, so we mapped them to full transcripts. The number of human sites that mapped to full transcripts was 42304, while for mouse the number of sites was 32940.

#### Negative dataset

To obtain non-methylated m^6^A sites, adenosines that conform to the DRACH motif were randomly selected from both mature mRNAs and full transcripts of the longest isoforms of Ensembl coding genes. Sites that overlapped with not only the positive samples, but also the curated previously identified human and mouse m6A peaks were removed.

#### Training and testing dataset

For human, 75% (29547) of all the positive sites that mapped to mature mRNAs and 75% (31728) of all the positive sites that mapped to full transcripts, along with the same number of negative samples were randomly selected as training datasets. The remaining 25% (9849 sites that mapped to mature mRNAs and 10576 that mapped to full transcripts) of all the positive sites were allocated to the independent testing datasets.

For mouse, the number of positive sites in training datasets was 22740 that mapped to mature mRNAs and 24705 that mapped to full transcripts. The mouse testing datasets concluded 7580 positive sites that mapped to mature mRNAs and 8235 positive sites that mapped to full transcripts.

Obviously, there are far more non-m^6^A sites than m^6^A sites across the transcriptome. Accordingly, an unbalanced 1:10 positive-to-negative ratio was maintained in our independent datasets for human and mouse (see S1–S8 Tables for these datasets). To build classifiers for mammalian m^6^A site prediction, the human and mouse training and testing datasets were joined.

### Features of RNAMethPre

For nucleotide sequence positioning around the adenosine sites, the mRNA sequence around the site was extracted and encoded as a binary vector according to a simple rule: ‘A’ -> 0001, ‘T’ -> 0010, ‘C’ -> 0100, and ‘G’ -> 1000. When the sites were located at the beginning or terminus of an mRNA, the gap character “N” was assigned to fill the sequence termini. Therefore, a *W*-nt flanking window of the sequence was encoded as a W*4-dimensional feature vector.

Nucleotide k-mer frequency was also considered. To represent the sequence context of an m^6^A/non-m^6^A site, the frequencies of all possible k-mer (k = 3, 4) nucleotides in a 101-nt flanking window centered around the sites were calculated.

With respect to the relative distribution of sites in transcripts, it has been reported that m^6^A sites are biased towards the 3’ ends of transcripts. Given a site, the absolute distance from the transcript start site was calculated and then scaled to obtain a relative position value (between 0 and 1).

Stability of the local structure was also considered. For each site, RNAFold [16] was used to fold the 101-bp mRNA fragment (from -50 to +50 with respect to the central *N*^6^-methyladenosine), yielding an MFE (minimum free energy) value. Then, the fragment sequence was shuffled 100 times and the MFE was calculated for each of the shuffled sequences. The *Z*-score of the MFE value for the original fragment was calculated to measure the secondary structure strength of the region harboring the site.

Finally, the feature vectors were combined and added to a single SVM.

### Support vector machine learning model training

SVM models were trained with the ‘RBF’ kernel function for the classifier. Parameters were optimized by a grid search on the training data. The SVM models were implemented in libsvm-3.21 [17].

### Evaluation of SVM prediction models

Seven-fold cross validation and independent tests were used to check the performance of our method with four frequently used measurements: specificity, sensitivity, AUROC (area under the ROC curve), and AUPR (area under the PR curve). The first three measurements were defined as follows:

$$Sensitivity=\frac{TP}{TP+FN}$$

$$Specificity=\frac{TN}{TN+FP}$$

$$MCC=\frac{TP\times TN-FP\times FN}{\sqrt{\left(TP+FP\right)\times \left(TP+FN\right)\times \left(TN+FP\right)\times \left(TN+FN\right)}}$$

where TP represents the number of true positive sites, defined as the correctly predicted m^6^A sites, TN represents the number of true negative sites, defined as the correctly predicted non-m^6^A sites, FP represents the number of false positive sites, defined as non-m^6^A sites predicted as m^6^A sites, and FN represents the number of false positive sites, defined as m^6^A sites predicted as non-m^6^A sites.

The ROC curve was obtained by plotting the false positive rate against the true positive rate at various threshold settings. The true positive rate is the same as sensitivity or recall, while the false positive rate can be calculated as (1-specificity). An area of 1.00 indicates a perfect predictor, and an area of 0.50 corresponds to a random model. The larger the area under the ROC curve, the more robust the model is. ROC curves can present an overly optimistic view of an algorithm’s performance if there is a skew of the dataset. To give a more informative picture of the predictors’ performance we introduced the area under precision-recall curve (AUPR). The Precision-Recall curves plot precision (the fraction of TP in all predicted positives) against recall (sensitivity) at various threshold settings. This curve is more sensitive to false positives than ROC curve.

To further test the sensitivity of our current predictor, the model was used to predict the curated previously identified m^6^A peaks from the literature [3, 4, 18, 19]. To identify specific methylated sites in known m^6^A peaks from m^6^A-seq, the sequences of the reported peaks were retrieved when the peak summit was reported, and a 200-bp flanking window centered around the peak summit was obtained. These peak sequences were added to our web server and the proportion of peaks that contain at least one predicted m^6^A site was calculated.

### Web Server construction

**Fig 1:**
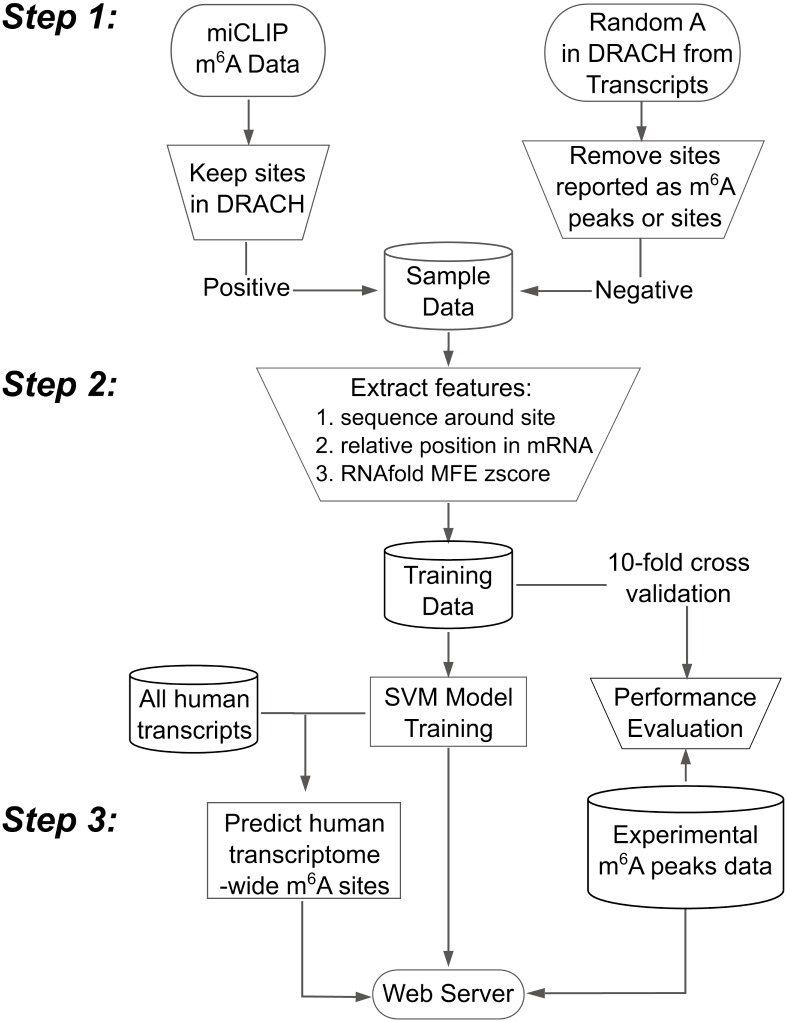
RNAMethPre Workflow. Positive and negative datasets were obtained (Step 1). Features of the datasets were extracted to obtain 366-dimensional vectors for each site as training data. The SVM classifier was trained to generate the SVM model and the performance of the model was evaluated (Step 2). Human transcriptome-wide m^6^A sites were predicted and a web server was constructed (Step 3).

PHP and SQLite were used to construct the RNAMethPre web-server, which implements the method described above. Given an mRNA and its corresponding species information, the server returns all predicted m^6^A sites to users. The results are also downloadable for further analysis. The SVM model was also applied to predict transcriptome-wide m^6^A sites. Experimental m^6^A-seq peaks were collected from the literature. The web server was built to provide both prediction and query services for m^6^A sites. A genome browser was also built based on JBrowse [20] to visualize the query results. Fig 1 illustrates the workflow for the development of RNAMethPre.

## Results and Discussion

### Model establishment

We built two prediction modes within our web server, i.e., the full transcript mode and the mature mRNA mode, consistent with SRAMP. Recent studies have shown that m^6^A exhibits both a nuclear role in pre-mRNA processing and a cytoplasmic role in the regulation of mRNA stability and translation, consistent with the findings that m^6^A occurs in both mature mRNA regions and introns [21, 22]. This suggests that the modification can be added at either the pre-mRNA level or the mature mRNA level, before or after RNA splicing. It is convenient to input either genomic sequences or mRNA sequences according to user needs. Therefore, both mature mRNA and full transcript modes were necessary. Classifiers for mammal, human, and mouse were built. The mammalian datasets included a combination of human and mouse datasets. We focus on the establishment of the predictor for mammal in both modes, as the human and mouse predictors were established in the same way.

**Fig 2:**
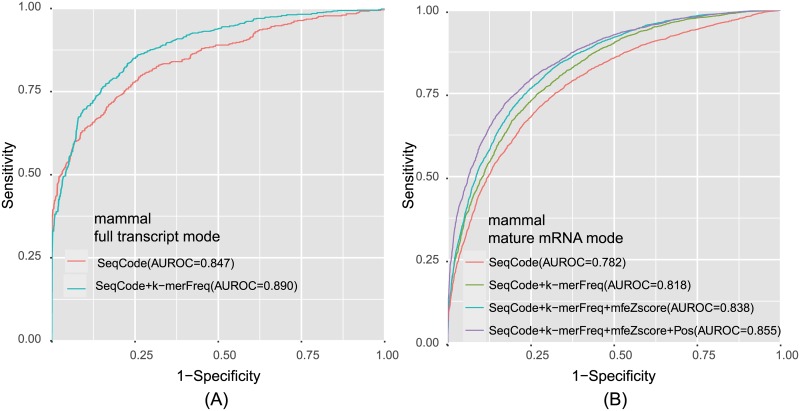
Overall Performances of Mammalian Classifiers Based on 5-fold Cross-validation Tests. (A) The ROC curve illustrating the performance for full transcript mode. (B) The ROC curve illustrating the performance for mature mRNA mode.

The positional binary specifying nucleotide sequence was used as the first feature to discriminate methylated DRACH motifs from un-methylated motifs. We optimized the length of the flanking window by building models for different sequence lengths. The optimized length was 11 nt (5 on each side of the focal sites) for mature mRNA mode and 31 nt (15 on each side of the focal sites) for full transcript mode. We trained the SVM classifier using the binary encoding and observed encouraging performance on the training dataset based on 5-fold cross-validation (Fig 2A; AUROC = 0.782, 0.847), indicating that the positional sequence pattern is a strong feature of m^6^A sites. It is notable that the flanking window length was shorter than that of SRAMP, but we achieved better performance on the training dataset by 5-fold cross-validation.

To illustrate the position-independent sequence pattern, we introduced the spectrum of k-mer nucleotide frequencies, which is widely employed to characterize bio-sequences [23, 24]. We calculated the k-mer (k = 3, 4) frequencies of 101-nt flanking windows centered around the methylated and non-methylated sites. The performance was improved substantially by incorporating the spectrum feature in the model training (Fig 2; AUROC = 0.818, 0.890), indicating this position-independent sequence feature indeed supplements the position-dependent encodings.

The AUROC for full transcript mode reached approximately 90%, but the AUROC for mature mRNA mode was unsatisfactory. To improve the performance of mature mRNA mode, we added two additional features to the model. In 2013, Schwartz et al. used nucleotide composition, local secondary structure stability, and relative position in the gene as features in their classifier to predict m^6^A sites in yeast and achieved promising performance results [11]. We applied the same strategy to incorporate the predicted secondary structure strength for each site and the distances from the transcription start and end sites to our model. Using these features, the AUROC increased to 85.5% for mature mRNA mode, but the full transcript mode showed little improvement. Therefore, we integrated all four features to the mature mRNA mode, but included only the two sequence features in the full transcript model (Fig 2).

In addition to the broad mammalian models, we built specific human and mouse predictors for both modes following the same procedure, and the predictors achieved good performance on the training dataset based on 5-fold cross-validation (S1 Fig). From S1 Fig, we can see that in full transcript mode, the AUROC of human and mouse are good and comparable. In mature mRNA mode, the AUROC of mouse is 0.924, which is far better than the AUROC of human (0.830). To check if the predictors for human and mouse sequences can be applied across species for either mode, we tested each predictor using independent datasets from the species. From the result shown in S9 and S10 Tables, we can see that in both modes, the performances of cross-species tests are lower than that of the intra-species tests, indicating the specificity of models for each species. As a result, based on the current dataset, we built unified predictors for mammals and species-specific predictors for human and mouse, which are all available in our RNAMethPre web server.

### Performance of the predictors on independent datasets

**Fig 3:**
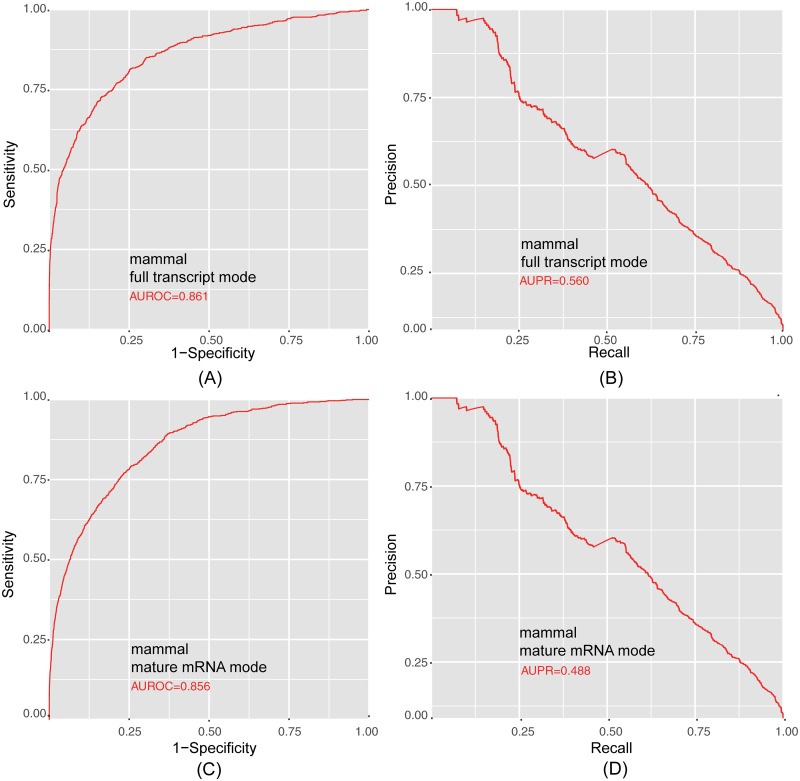
Performances of the Mammalian Classifiers on Independent Testing Datasets. (A) ROC curve illustrating the performance on the unbalanced independent testing dataset in full transcript mode. (B) Precision-recall curve illustrating the performance on the unbalanced independent testing dataset of full transcript mode. (C) ROC curve illustrating the performance on the unbalanced independent testing dataset of mature mRNA mode. (D) Precision-recall curve illustrating the performance on the unbalanced independent testing dataset of mature mRNA mode.

In order to validate the method, we tested the model on independent datasets. Since there are far more non-methylated sites than methylated sites in the transcriptome, we set the ratio of m^6^A sites to non-m^6^A sites to 1:10 in our independent datasets. The results of the independent tests generally agreed well with those from the cross-validation tests. The full transcript mode and mature mRNA mode models for mammal achieved AUROCs of 0.886 and 0.856, respectively (Fig 3). For more precise analyses of performance, we applied four stringency thresholds corresponding to 90%, 85%, and 80% specificities in the independent dataset tests (Table 1).

**Table 1:** Performance of RNAMethPre for various stringency thresholds and comparison with SRAMP.

Predictor	Confidence	Specificity	Sensitivity		MCC	
			Rnamethpre	Sramp	Rnamethpre	Sramp
Mature mRNA mode	High	90.0%	46.8%	44.0%	0.311	0.293
	Moderate	85.2%	56.0%	54.2%	0.305	0.294
	Low	80.0%	63.8%	-	0.298	-
Full transcript mode	High	93.0%	64.0%	50.3%	0.496	0.405
	Moderate	88.0%	74.0%	64.5%	0.465	0.385
	Low	83.0%	81.0%	72.8%	0.435	0.414

Moreover, we tested the model on previously identified human m^6^A peaks for which specific methylated sites were not assigned. We identified specific m6A sites in previously detected peaks using the mature mRNA mode since almost all of the peaks are located in mature mRNA. And we chose the moderate threshold during the prediction. For human, low-resolution human m6A data were downloaded from literature, yielding 389282 m6A peaks of 25 different tissues and conditions, ranging from 100 to 200 bp in length. After removing peaks that did not contain the DRACH motif, 310423 peaks were retained for performance analyses. We found that 68.1% (211403 peaks) of the peaks were predicted to be an m6A site by human predictor, demonstrating that our method is sensitive. For mouse, 207971 peaks were downloaded. Among the 176770 peaks with at least one DRACH motif, 70.4% were predicted to contain at least one m6A site by the mouse predictor. What should also be noticed is that a considerable fraction (51% for human and 53% for mouse) of the peaks were predicted to harbor multiple m6A sites. This observation is quite consistent with the previous report that multiple m6A sites can appear in clusters and may be detected underneath the same m6A peak [4].

To further evaluate the performance of RNAMethPre, we compared it with the predictors in the recently developed web server SRAMP [14]. Since SRAMP only includes mammalian predictors, the comparison was limited to mammals for both prediction modes. As shown in Table 2, AUROC and AUPR for RNAMethPre were comparable to those of SRAMP for full transcript mode. However, for mature mRNA mode, AUROC of RNAMethPre was 5% higher and AUPR was 6% higher than those of SRAMP. The sensitivity and MCC of RNAMethPre were also higher than those of SRAMP for the same specificities in either mode (Table 1). These results clearly indicated that the performance of RNAMethPre is superior to that of its counterpart in predicting methylated sites of mRNA

**Table 2:** Comparison of RNAMethPre with the Existing Web Server SRAMP using Independent Unbalanced Datasets.

Predictor	Mode	AUROC	AUPR
RNAMethPre	full transcript	0.886	0.560
SRAMP	full transcript	0.891	0.523
RNAMethPre	mature mRNA	0.856	0.488
SRAMP	mature mRNA	0.797	0.312

### Application of the method to identify transcriptome-wide m^6^A sites

A limited number of experiments have identified m^6^A peaks or sites using high-throughput methods, but these do not capture all m^6^A sites because the m^6^A modification is dynamic and tissue-specific. Here, we applied RNAMethPre to identify all potential sites that can be methylated across the human transcriptome using a moderate confidence threshold in mature mRNA mode. A total of 203106 confident m^6^A sites were identified for 58939 human mRNA sequences (refSeq, hg19). For mouse, 267521 confident m^6^A sites were identified for 35842 mRNA sequences (refSeq, mm9). The prediction results uncovered all potential m^6^A sites across the transcriptome and provided a powerful supplement to current high-throughput data. All these predicted sites are available for query or download on our web server.

We also applied the prediction method to assign specific methylated sites in previously identified m^6^A-seq peaks. Typically, m^6^A-seq peaks are 100–200 nt, and previous identifications of m^6^A residues are limited to one site per peak, i.e., the site in the consensus motif that is nearest to the peak summit or center. This approach misses a substantial portion of clustered m^6^A sites. However, it is not appropriate to classify all sites in DRACH motifs in peaks as methylated, since not all DRACH motifs are methylated. We identified specific m^6^A sites in previously detected peaks, and a considerable fraction of the peaks were predicted to harbor multiple m^6^A sites.

### Web server

**Fig 4:**
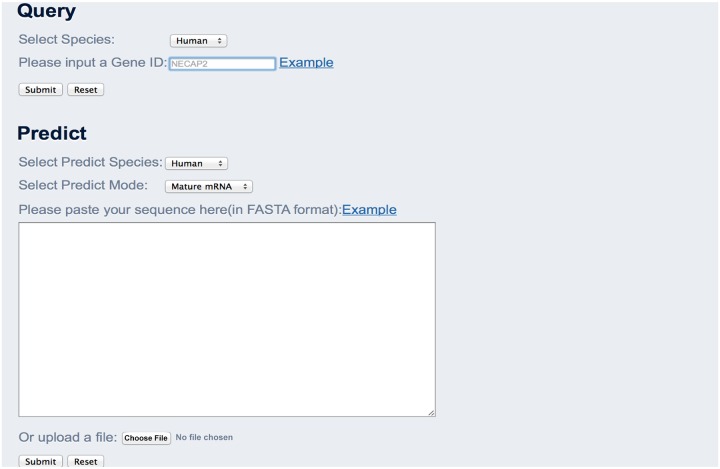
The user interface of the RNAMethPre web server.

As described on the home page of RNAMethPre (Fig 4), the web server contains two parts: “Query” and “Predict.” In the “Predict” section, the input is the RNA or DNA sequence in FASTA format. Users have the option to choose the full transcript mode or the mature mRNA mode, for genomic or mature mRNA sequence data. Users can select the taxon, i.e., human, mouse, or mammal, as appropriate. The results table reports the ID of the input sequence, position of the predicted site, flanking sequence, and prediction threshold. A link to download the prediction results is provided on the top of the results table. The prediction speed is fast. Therefore, RNAMethPre was suitable for batch operation; users can submit more than one mRNA sequence for prediction simultaneously and obtain fast prediction results.

**Fig 5:**
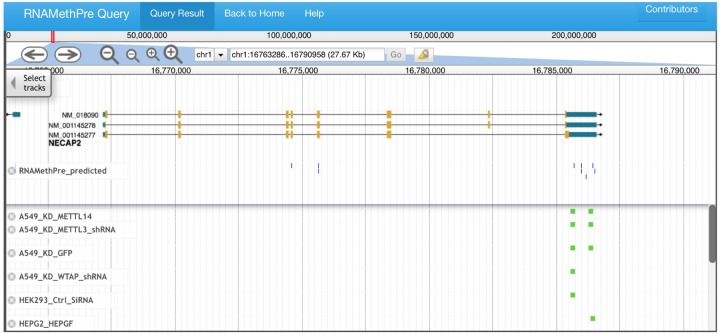
The genome browser to visualize the query results.

The database of predicted as well as experimentally determined high-throughput m^6^A sites or peaks across the whole transcriptome is built at the back end of the server. For the predicted sites part, there are 443485 human m6A sites and 406519 mouse m6A sites. For the experimentally peaks part, the database contains 25 m6A peaks datasets of human and 18 m6A peak datasets of mouse which are the m6A peaks identified in different tissues or conditions. Accordingly, in the “Query” section, users can view the m^6^A sites in a queried gene within seconds. In addition to the detailed results table describing each predicted m^6^A site in the query gene, a genome browser based on JBrowse [20] was built to visualize all query results (Fig 5). In the browser, users can select a particular tissue type and can conveniently check predicted or experimental m^6^A sites and peaks.

### Conclusion

RNAMethPre employed SVM methods to build classifiers to predict m^6^A modifications of mammalian mRNA and was effective for both full transcript mode and mature mRNA mode. The web server is user-friendly and comprehensive, providing not only a highly efficient m^6^A prediction service, but also a database of predicted m^6^A sites as well as experimental m^6^A sites and peaks across the transcriptome for query and visualization. Future developments include the improvement of performance by incorporating more effective features and the support of data from additional species with single-nucleotide resolution m^6^A sites. RNAMethPre provides a basis for understanding the broad functional effects and general properties of m^6^A modifications.

## Supporting Information

S1 Fig The overall performances of the human and mouse classifiers based on the results from 5-fold cross-validation tests. (DOCX)

S2 Fig The performances of the human SVM classifiers on the independent unbalanced testing datasets. (DOCX)

S3 Fig The performances of the mouse SVM classifiers on the independent unbalanced testing datasets. (DOCX)

S1 Table The human training dataset for the full transcript mode predictor. (XLSX)

S2 Table The human testing dataset for the full transcript mode predictor. (XLSX)

S3 Table The human training dataset for the mature mRNA mode predictor. (XLSX)

S4 Table The human testing dataset for the mature mRNA mode predictor. (XLSX)

S5 Table The mouse training dataset for the full transcript mode predictor. (XLSX)

S6 Table The mouse testing dataset for the full transcript mode predictor. (XLSX)

S7 Table The mouse training dataset for the mature mRNA mode predictor. (XLSX)

S8 Table The mouse testing dataset for the mature mRNA mode predictor. (XLSX)

S9 Table The cross-species prediction performance for full transcript mode. (DOCX)

S10 Table The cross-species prediction performance for mature mRNA mode. (DOCX)
