# Supplementary material for: RNAMethPre: A Web Server for the Prediction and Query of mRNA m6A Sites
Source: PLoS One. 2016 Oct 10;11(10):e0162707. doi: 10.1371/journal.pone.0162707 (PMC5056760; doi:10.1371/journal.pone.0162707)
Supplement: S1 Fig — (DOCX) [file pone.0162707.s001.docx]

**S1 Fig.** The overall performances of the human and mouse classifiers based on the results from 5-fold cross-validation tests. (A) The ROC curve illustrating the performances of human SVM models for full transcript mode. (B) The ROC curve illustrating the performances of human SVM models for mature mRNA mode. (C) The ROC curve illustrating the performances of mouse SVM models for full transcript mode. (D) The ROC curve illustrating the performances of mouse SVM models for mature mRNA mode.
