# Supplementary material for: RNAMethPre: A Web Server for the Prediction and Query of mRNA m6A Sites
Source: PLoS One. 2016 Oct 10;11(10):e0162707. doi: 10.1371/journal.pone.0162707 (PMC5056760; doi:10.1371/journal.pone.0162707)
Supplement: S3 Fig — (DOCX) [file pone.0162707.s003.docx]

**S3 Fig.** The performances of the mouse SVM classifiers on the independent unbalanced testing datasets. (A) The ROC curve illustrating the performance on the unbalanced independent testing dataset of mouse in mature mRNA mode. (B) The PR curve illustrating the performance on the unbalanced independent testing dataset of mouse in mature mRNA mode. (C) The ROC curve illustrating the performance on the unbalanced independent testing dataset of mouse in full transcript mode. (D) The PR curve illustrating the performance on the unbalanced independent testing dataset of mouse in full transcript mode.
