# Supplementary material for: RNAMethPre: A Web Server for the Prediction and Query of mRNA m6A Sites
Source: PLoS One. 2016 Oct 10;11(10):e0162707. doi: 10.1371/journal.pone.0162707 (PMC5056760; doi:10.1371/journal.pone.0162707)
Supplement: S9 Table — (DOCX) [file pone.0162707.s012.docx]

| Species | Human (model) | Mouse (model) | Mammal (model) |
| --- | --- | --- | --- |
| Human (testData) | 0.893 | 0.852 | 0.869 |
| Mouse (testData) | 0.871 | 0.940 | 0.884 |
| Mammal (testData) | 0.880 | 0.876 | 0.901 |

**S9 Table**. The cross-species prediction performance for full transcript mode.
