# Supplementary material for: RNAMethPre: A Web Server for the Prediction and Query of mRNA m6A Sites
Source: PLoS One. 2016 Oct 10;11(10):e0162707. doi: 10.1371/journal.pone.0162707 (PMC5056760; doi:10.1371/journal.pone.0162707)
Supplement: S10 Table — (DOCX) [file pone.0162707.s013.docx]

| Species | Human (model) | Mouse (model) | Mammal (model) |
| --- | --- | --- | --- |
| Human (testData) | 0.842 | 0.664 | 0.739 |
| Mouse (testData) | 0.816 | 0.920 | 0.891 |
| Mammal (testData) | 0.828 | 0.834 | 0.863 |

**S10 Table**. The cross-species prediction performance for mature mRNA mode.
